# Innovative prevention and control of coccidiosis: targeting sporogony for new control agent development

**DOI:** 10.1016/j.psj.2024.104246

**Published:** 2024-08-22

**Authors:** Dan Zhao, Jingxia Suo, Lin Liang, Ruiying Liang, Rongqiong Zhou, Jiabo Ding, Xianyong Liu, Xun Suo, Sixin Zhang, Xinming Tang

**Affiliations:** ⁎ Key Laboratory of Animal Biosafety Risk Prevention and Control (North) & Key Laboratory of Veterinary Biological Products and Chemical Drugs of MARA, Institute of Animal Science, Chinese Academy of Agricultural Sciences, Beijing 100193, China; † College of Veterinary Medicine, Southwest University, Chongqing, 400715, China; ‡ National Key Laboratory of Veterinary Public Health Security, Key Laboratory of Animal Epidemiology of the MARA, National Animal Protozoa Laboratory & College of Veterinary Medicine, China Agricultural University, Beijing 100193, China

**Keywords:** chicken coccidiosis, life cycle, sporogony, developmental regulation, control agent

## Abstract

Coccidiosis is one of the most significant diseases affecting the poultry industry, with recent estimates indicating that it causes annual losses exceeding £10 billion globally. Increasing concerns over drug residues and resistance have elevated the importance of safe and effective vaccines as the primary method for controlling coccidiosis and other animal diseases. However, current commercial live vaccines for coccidiosis can negatively impact the feed conversion rates of young broilers and induce subclinical symptoms of coccidiosis, limiting their widespread adoption. *Eimeria* species, the causative agents of coccidiosis, exhibit unique biological characteristics. Their life cycle involves 2 or more generations of schizogony and 1 generation of gametogony within the host, followed by sporogony in a suitable external environment. Sporogony is crucial for *Eimeria* oocysts to become infectious and propagate within the host. Focusing on the sporogony process of *Eimeria* presents a promising approach to overcoming technical challenges in the efficient control of coccidiosis, addressing the urgent need for sustainable and healthy farming practices. This paper systematically reviews existing control strategies for coccidiosis, identifies current challenges, and emphasizes the research progress and future directions in developing control agents targeting sporogony. The goal is to provide guidance for the formulation of scientific prevention and control measures for coccidiosis.

## Introduction

Coccidiosis, caused by *Eimeria* species, is a common disease in the livestock industry, affecting many animals and posing a significant threat to the poultry industry in particular (Liu et al., 2023). As the intensive livestock industry rapidly scales up, controlling coccidiosis in poultry farms has become increasingly challenging. Once infectious oocysts are released and spread, they can cause outbreaks of coccidiosis, leading to severe economic losses (Gao et al., 2024).

Currently, poultry farms primarily control coccidiosis using vaccines, drugs, and improved management practices (Attree et al., 2021). Disinfectants are used less frequently; those with low efficacy are almost ineffective, while stronger disinfectants can significantly impact both humans and livestock (You, 2014). Appropriate vaccines can significantly reduce the incidence of coccidiosis and related economic losses, but improper use can lead to virulent reversion or disease outbreaks (Wang and Suo, 2020). Traditional antibiotic control has faced extensive failure due to misuse, resulting in the emergence of multidrug-resistant strains and drug residues in animal-derived products, adding further challenges to controlling coccidiosis in farms.

From the entry of *Eimeria* oocysts into the chicken's digestive system to their excretion and subsequent reacquisition of infectivity, the most critical stage is sporulation. Interrupting the sporulation process or eliminating unsporulated oocysts before they become infectious can significantly reduce the spread of coccidiosis. Therefore, the sporulation stage is crucial for effective prevention and control of the disease. Based on a summary of traditional coccidiosis prevention and control measures, this paper systematically reviews various formulations and their effects targeting the neglected stage of sporogony, proposing new strategies for more effective coccidiosis prevention and control.

### Impact of Coccidiosis on Chicken Health

Chicken coccidiosis does not exhibit distinct seasonality, and chickens of all ages are susceptible. The occurrence of coccidiosis is often associated with various factors, including management practices, environmental conditions, and the breed and age of the chickens. Coccidiosis is primarily transmitted orally through contaminated oocysts in the environment. Oocysts can contaminate litter, cages, feeding tools, or drinking water. When chickens ingest infectious oocysts, the parasites undergo asexual and sexual reproduction *in vivo*. The resulting offspring oocysts are excreted in the feces, releasing a large number of oocysts and causing widespread infection in the flock. Additionally, oocysts can remain infectious in the environment for several months (You, 2014). Therefore, once coccidiosis occurs, it is difficult to eradicate it in a short period of time.

The well recognized 7 species of the genus *Eimeria* in chickens include *E. tenella, E. maxima, E. acervulina, E. praecox, E. brunetti, E. necatrix* and *E. mitis*. However, the discovery of 3 cryptic *Eimeria* operational taxonomic units (**OTU**) in chickens across various continents has garnered significant interest in recent years. These strains, distinguished by their genotypic and phenotypic characteristics, have been classified as distinct parasite species and named *E. lata, E. nagambie*, and *E. zaria* (Blake et al., 2021b). The differentiation of various *Eimeria* species primarily depends on factors such as the site of parasitism, pathogenicity, oocyst morphology, minimum prepatent period, and the shortest sporulation time (Gao et al., 2024). Additionally, the more precise PCR and quantitative real-time PCR method for distinguishing between different species have been successfully established (Vrba et al., 2010). The lack of cross-immune protection or very weak cross-immune protection among different species is another method of differentiation. However, due to the significant variation in cross-immunogenicity among different isolates, this method is often not suitable for identifying *E. maxima* (Jenkins et al., 2024).

*Eimeria* parasites primarily causes intestinal diseases in poultry. Acute symptoms include depression, ruffled feathers, persistent diarrhea, anemia and pallor in combs and visible mucous membranes, and in severe cases, bloody feces, with droppings appearing orange-red. Due to extensive damage to intestinal epithelial cells and increased autointoxication, affected birds may experience ataxia, coma, convulsions, or death (Zhou et al., 2023). Chicks with low immunity may die within 7 d of infection, with mortality rates as high as 80% (Liu et al., 2023). Chronic symptoms include reduced appetite, weight loss or stunted growth, intermittent diarrhea, decreased feed conversion rates, and lower egg production in laying hens. The risk of mixed infections increases, as coccidiosis can induce *Clostridium perfringens* infections leading to necrotic enteritis (Antonissen et al., 2016; Lu et al., 2020; Feng et al., 2022). Affected birds show signs of depression, reduced appetite, anemia, pale wattles, severe diarrhea with red or black tar-like feces, sometimes containing intestinal mucosal tissue. Secondary infections with *Escherichia coli* and Newcastle disease are common, causing high fever, respiratory distress, severe diarrhea, neurological disorders, and hemorrhaging in mucous and serous membranes (Butt et al., 2019). Additionally, reovirus and Marek's disease virus can increase the infection rate of coccidiosis, severely impacting poultry health and production capacity (El-Shall et al., 2022).

### Life Cycle of *Eimeria*

The life cycle of *Eimeria* consists of 3 stages: schizogony, gametogony, and sporogony. Schizogony and gametogony are endogenous reproductive processes, while sporogony is an exogenous reproductive process. When poultry inadvertently ingest infectious (sporulated) oocysts, the oocysts first enter the gizzard, where the initial sporozoite release occurs. The wall of the oocyst breaks down due to enzymatic digestion and mechanical grinding, releasing sporocysts. The sporocysts are further released in the small intestine under the action of trypsin, and the subsequent sporozoites are transported to the site of parasitism of each species, where they invasion and develop within the intestinal epithelial cells or intestinal gland cells, such as during certain developmental stages of *E. necatrix* (Burrell et al., 2020).

### Schizogony

**Figure 1:**
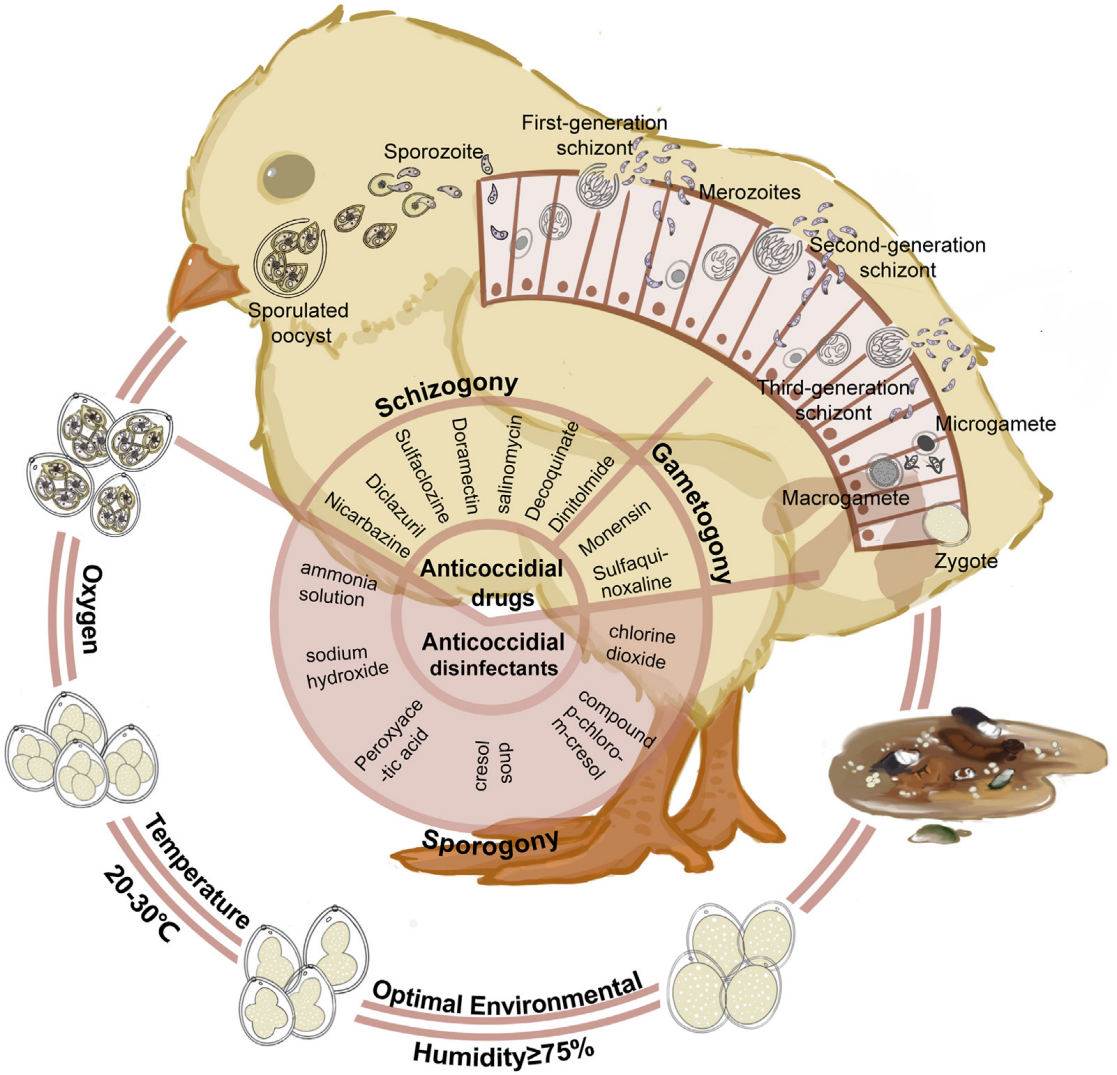
Lifecycle of *Eimeria* and Anticoccidial Control Strategies in Chickens. The lifecycle of *Eimeria* spp., the causative agents of coccidiosis in chickens, comprises 3 main stages: sporogony, schizogony, and gametogony. Specific anticoccidial drugs or disinfectants target different stages of *Eimeria's* development. Sporogony: Unsporulated oocysts are shed in feces and become infectious in the environment. Optimal conditions for sporulation include oxygen, a temperature of 20°C to 30°C, and humidity levels of ≥75% in feces. Disinfectants effective at this stage include ammonia solution, sodium hydroxide, peracetic acid, and cresol soap. Schizogony: Within the host, ingested sporulated oocysts release sporozoites that invade intestinal cells. The sporozoites undergo multiple rounds of asexual reproduction, forming first-generation schizonts, followed by merozoites, second-generation schizonts, and so on. Anticoccidial drugs effective at this stage include sulfaquinoxaline, sulfaclozine, diclazuril, nicarbazine, doramectin, salinomycin, decoquinate, dinitolmide, and monensin. Gametogony: Merozoites differentiate into sexual stages, forming microgametes (male) and macrogametes (female), which fuse to form zygotes that develop into oocysts. Anticoccidial drugs effective at this stage include chlorine dioxide, compound p-chloro-m-xylenol, and cresol soap.

Schizogony, also known as asexual reproduction, is primarily completed through multiple divisions (typically 3-4 times). Using *E. tenella* as an example, when sporozoites reach the cecum, they first invade the surface epithelial cells and then proceed to the lamina propria. The parasites that invade the epithelial cells quickly develop into schizonts (Figure 1). Schizonts undergo multiple nuclear divisions to produce the first-generation merozoites, which are banana-shaped and are released 2-3 days post infection, destroying host cells in the process (Mesa-Pineda et al., 2021). These merozoites invade new intestinal epithelial cells and divide into second-generation merozoites, further damaging the intestine. *Eimeria* undergoes 2 rounds of schizogony in a single reproductive cycle. This process repeats within the host, producing an increasing number of progenies. Schizogony causes the most severe intestinal damage during the entire life cycle, as merozoites shuttle back and forth, destroying many intestinal mucosal epithelial cells, thickening the cecal wall, causing severe hemorrhage, and impairing nutrient absorption (Nabian et al., 2018). This also sets the stage for secondary infections.

### Gametogony

Gametogony, also known as sexual reproduction, involves the formation and fusion of male and female gametes to form a zygote. Most merozoites develop into microgametes (male gametes) and macrogametes (female gametes) after the second division, with this process also occurring within epithelial cells (Walker et al., 2013). Microgametes further develop flagella, gaining motility and the ability to leave host cells and move within the intestine. Microgametes enter cells containing macrogametes and fuse with mature macrogametes to form zygotes (Figure 1). Studies have found that GAM56 and GAM82 proteins in gametocytes can act as protective antigens, stimulating an immune response. For instance, a DNA vaccine using GAM56 as antigen target showed good immune protection (Xu et al., 2013).

### Sporogony

Sporogony refers to the process where zygotes further develop into oocysts and then mature sporozoites in the external environment (Rieux et al., 2012). During gametogony, microgametes and macrogametes form zygotes, which then develop thick walls and become oocysts (Jonscher et al., 2015). At this stage, the oocysts are not yet infectious. Once excreted in feces, the oocysts continue to develop under suitable humidity and temperature, with warm and humid conditions accelerating their development (Figure 1). However, too wet litter will reduce sporulation due to insufficient oxygen (Waldenstedt et al., 2001). The minimum sporulation time is varying among different species of *Eimeria*. Under optimal conditions, the sporulation rate of chicken coccidia generally exceeds 90% within 48 hours. Upon completing sporulation, the oocysts contain 4 sporocysts, each with 2 sporozoites, and develop a thick wall that confers strong environmental resistance, making the oocysts infectious. Since sporogony occurs outside the host and infectivity depends entirely on the completion of sporulation, using insecticides to kill unsporulated oocysts or using drugs and inhibitory conditions to stop the sporulation process can significantly reduce the spread of coccidiosis. For instance, oocysts are relatively weak against high temperatures and dryness (Attree et al., 2021). Sporulation of coccidia oocyst ceases at 40°C, and oocysts are killed at around 66°C. If litter is treated at 40°C for 3-5 days, the oocysts can be completely inactivated, thereby reducing the spread of chicken coccidiosis (Jenkins et al., 2023).

## Coccidiosis Vaccines and Their Mechanisms of Action

### Live Vaccines for Coccidiosis

Despite the ongoing development and commercialization of various coccidiosis vaccines, live vaccines remain a critical method for preventing coccidiosis in chickens, surpassing live vector vaccines and recombinant vaccines in importance. Live vaccines contain either attenuated or unmodified *Eimeria* parasites, which have reduced pathogenicity and do not cause severe disease but can still trigger an immune response (Blake et al., 2021a). The protective mechanism of live coccidian vaccines was studied using mice model, revealing a close association with resident memory CD8^+^ T cells. This finding offers valuable insights for the development of vaccines against other protozoan parasites (Shi et al., 2023). However, the reproductive capacity, pathogenicity, and sites of infection within the intestine vary among different *Eimeria* species. Consequently, the number of parasites and frequency of infection needed to induce sufficient immunity largely depend on the specific *Eimeria* species. Additionally, factors such as the timing of immunization, chicken breed, and environmental conditions can influence vaccine efficacy (Soutter et al., 2020). Studies have shown that vaccination with 1 strain does not confer cross-protection against different species or even different strains of the same species (Blake et al., 2011). To ensure comprehensive protection, chickens must be vaccinated with oocysts corresponding to each *Eimeria* species, which promotes the use of multivalent vaccines. Future research should focus on this area and the discovery of new antigens to expedite vaccine development (Zaheer et al., 2022).

#### Virulent Vaccines

Virulent vaccines are prepared from strains isolated from naturally infected chickens or their feces. These strains are typically collected using saturated saline flotation and purified through single oocyst isolation, then mixed with an appropriate stabilizer. Upon initial infection, the parasites reproduce within the chickens, maintaining a low-level infection without causing disease. New oocysts are excreted and sporulate under suitable temperature and humidity conditions. Chickens can acquire immunity by ingesting infectious oocysts from litter, resulting in high immune protection (Attree et al., 2021). However, the strong pathogenicity of virulent vaccines necessitates strict dose control to prevent introducing wild strains into the farm or causing widespread coccidiosis. Additionally, when using coccidiosis vaccines during rearing, oocysts can quickly settle in water, leading to uneven mixing or flocculation, affecting immunization efficacy. Therefore, there are certain risks and limitations associated with virulent vaccines. They may also cause mild symptoms in chickens, reducing feed conversion rates and slowing growth (Zheng et al., 2023).

#### Attenuated Vaccines

Attenuated vaccines are live vaccines prepared by artificially weakening the virulence of virulent strains isolated from the field through techniques such as continuous precocious selection, physical and chemical treatment, cell culture, and continuous passage in chicken embryo (Liu et al., 2023). The pathogenicity of attenuated vaccine is significantly reduced, but retains good immunogenicity (Tewari and Maharana, 2011). The attenuated strains can cause mild infections during vaccination, stimulating the host's immune system to produce antibodies and immune cells, providing protection without causing severe intestinal damage. The production process for attenuated vaccines is more complex. Additionally, although the strains in the vaccine have reduced pathogenicity, there are still some safety concerns, such as causing mild adverse reactions. Therefore, stability and safety are critical considerations during vaccine production.

### Transmission-Blocking Subunit Vaccine

Anticoccidial vaccine composed of protective antigens, either native or recombinant, has been developed as an alternative to live vaccines to address the associated issues and reduce costs. CoxAbic is a transmission-blocking vaccine that utilizes antigens derived from the wall-forming bodies of *Eimeria* maxima macrogametocytes. These antigens are crucial in forming the resilient oocyst wall, which protects the parasite in the environment (Sharman et al., 2010). By targeting these antigens, CoxAbic interferes with the oocyst formation, thereby reducing the spread of the parasite. The vaccine is administered to breeder hens, resulting in the transfer of protective maternal antibodies to their offspring via the egg yolk, providing early and effective immunity against coccidiosis. The development of CoxAbic marks a pivotal advancement in the field of veterinary vaccines, showcasing the potential of maternal immunization strategies. This approach not only ensures early protection of the hatchlings but also minimizes the stress and logistical challenges associated with vaccinating young chicks (Titilincu et al., 2005). Field trials conducted in various countries have demonstrated the vaccine's efficacy and safety, with significant reductions in oocyst shedding and coccidial lesions observed in vaccinated flocks (Wallach et al., 2008). As the demand for chemical-free poultry products grows, vaccines like CoxAbic offer a promising solution for sustainable and effective coccidiosis control. Future research is likely to focus on improving the production efficiency of the vaccine, possibly through recombinant antigen technologies, to reduce costs and enhance accessibility. The success of CoxAbic paves the way for developing similar vaccines targeting other stages of the parasite's lifecycle or other apicomplexan parasites affecting livestock (Sharman et al., 2010).

### Novel Genetic Engineering Vaccines

Genetic engineering vaccines employ recombinant DNA technology to insert natural or synthetic genetic material into bacteria, yeast, or animal cells. These cells then clone and express the desired antigen, which is purified to create the vaccine. This category includes subunit vaccines, vector vaccines, nucleic acid vaccines, and gene-deleted live vaccines (Cid and Bolívar, 2021).

#### Discovery of Protective Antigens in *Eimeria*

##### Microneme Proteins

It is currently believed that there are over 20 microneme proteins in *Eimeria*, but only a few genes have been functionally annotated and studied. Microneme protein 1 (EtMIC-1) and microneme protein 2 (EtMIC-2) are both involved in the parasite's invasion of host cells (Ding et al., 2005). EtMIC-2 is closely linked to the immune protection against coccidiosis in chickens. EtMIC-1 is a glycoprotein G-related protein mainly expressed during the merozoite and sporozoite stages, while EtMIC-2 is an acidic protein expressed in sporozoites and first- and second-generation merozoites, typically during the parasite's invasion of host cells (Tomley et al., 1996; Sun et al., 2014). The tissue lesions in chickens immunized with EtMIC-2 expressed in *E. coli* were significantly reduced after parasite challenge (Yan et al., 2018). Additionally, the expression of EtMIC-2 protein in *Pichia pastoris* demonstrated significant immunogenicity, stimulating a robust immune response in the host. The protection of EtMIC-2 protein in *P. pastoris* was stronger compared to *E. coli* (Zhang et al., 2014). Although the *E. coli* expression system is more commonly used in experiments, *P. pastoris*, as a eukaryote, possesses the capability for post-translational modifications, resulting in a protein that more closely resembles its natural structure (Francis, 2018).

##### Refractile Body Proteins

Refractile body proteins are highly immunogenic and appear during the sporozoite stage, disappearing during schizogony, thus being associated with parasite invasion. The most representative of these is SO7 (GX3262), whose product can induce an immune response in chickens, providing protective effects. A study used *E. coli* expressing the recombinant nonantibiotic SO7 gene to orally immunize 3-day-old chicks, followed by a booster immunization 2 weeks later. Comprehensive measurements indicated that it effectively stimulated a protective immune response in the host (Yang et al., 2010). In addition, vaccines designed with recombinant *Eimeria* SO7 protein could effectively induce both humoral and cellular immunity in chickens (Rafiqi et al., 2018). Additionally, the refractile body protein Etp28 has been found to be expressed at different stages of the parasite's life cycle and exhibits immunogenicity, inhibiting merozoite activity (Yang et al., 1998). Understanding its mechanism of action could aid vaccine research, enhancing the prevention and control of coccidiosis in chickens, making it a current research focus.

##### Apical Membrane Antigen

Apical membrane antigen-1 (**AMA-1**) is a conserved protein secreted by the micronemes to the parasite surface, playing a crucial role during *Plasmodium* invasion of host cells and being associated with *Toxoplasma gondii* intracellular proliferation (Jahangiri et al., 2019). It is considered one of the most promising malaria vaccine candidate antigens. AMA-1 has been found in various protozoa, but research on coccidia AMA-1 remains unclear. An experiment was conducted to evaluate the protective effect of the AMA-1 antigen against *Eimeria* infection by developing a vaccine based on the AMA-1 antigen (Fatoba and Adeleke, 2020). The results showed that the immunized group had better overall health, less cecal damage, and significantly lower oocyst output compared to the control group, indicating that AMA-1 could provide some degree of immune protection against infection, making it a valuable candidate antigen for vaccines (Blake et al., 2011).

##### Rhomboid Proteins

Rhomboid proteins (ROMs) are a class of intramembrane serine proteases involved in epidermal growth factor and its receptor signal transduction (Dowse and Soldati, 2005). Research shows that these proteases are present in *Plasmodium, Toxoplasma,* and *Eimeria*, though their biological functions differ among species. Zheng and colleagues found that ROM3 in *E. tenella* is primarily involved in cleaving EtMIC4 (Zheng et al., 2014). Eukaryotic expression products of EmROM3 were used for muscle injection immunization in chickens. Subsequent measurements of serum IgG antibody levels and transcription levels of related cytokines were conducted to evaluate induced humoral and cellular immune responses. The results showed partial protective effects against *E. maxima*, indicating that ROM play an important role during the parasite's invasion of host cells (Chen et al., 2021a).

##### Cross-Immune Protection

Cross-immune protection refers to the ability of an organism's immune response to a specific pathogen to provide protection other or different serotypes. This concept is crucial in vaccine design and disease control because it can broaden the vaccine's protective range, allowing a single vaccine to combat multiple pathogens. Consequently, multivalent vaccines have emerged as highly advantageous over monovalent vaccines, as they can prevent multiple strains of coccidiosis with a single immunization, saving time and effort. Researchers discovered that the constructed monovalent or multivalent vaccines targeting *E. tenella, E. necatrix, E. maxima*, and *E. acervulina* not only provided protection against the corresponding species, but also induced partial cross immunity protection (Song et al., 2009, 2015b).

Additionally, studies have shown that multivalent recombinant vaccines produced using the *E. coli* expression system possess certain immunoprotective effects. The *P. pastoris* expression system, the second most commonly used protein expression system after *E. coli*, is also widely used in experiments. This vaccine demonstrated improved immunoprotection in clinical trials. Song and colleagues designed a multivalent subunit vaccine containing 4 recombinant proteins from different *Eimeria* species: *E. necatrix* NA4 antigen, *E. tenella* TA antigen, *E. maxima* Em8 antigen, and *E. brunetti* LDH antigen. This vaccine provided comprehensive protection for chicks against multiple *Eimeria* infections (Song et al., 2015a). Although multi-component recombinant vaccines have demonstrated promising protective capabilities during the laboratory research stage, challenges such as reliance on adjuvants, the precise balance and dosage of various antigen components, and the necessity for repeated immunizations pose significant hurdles for their commercialization and practical application in the field. Addressing these issues necessitates innovative advancements in both theoretical understanding and technological approaches.

#### Application of Adjuvant Molecules

Expressing molecular adjuvants are biomolecules used to enhance the immunogenicity of vaccines, thereby increasing their effectiveness. For example, Bacille Calmette-Guérin (**BCG**) is a live attenuated vaccine used to prevent tuberculosis, and it can serve as an adjuvant to boost the immune response. BCG as a vaccine carrier shows promising potential in current vaccine development. Wang *et al.* developed a novel recombinant BCG vaccine incorporating the diamond-shaped protein gene and the IL-2 gene, administered via nasal and subcutaneous injections. The results demonstrated high CD4^+^ and CD8^+^ T cell ratios, reduced cecal lesion scores, and increased weight gain, indicating robust immune efficacy (Wang et al., 2014).

Researchers constructed a transgenic *Eimeria* line that stably expresses IgY Fc and evaluated its impact on immunogenicity through controlled experiments. The results indicated that transgenic *Eimeria* expressing IgY Fc offers significant advantages, and IgY Fc as a molecular adjuvant can enhance the immunogenicity of vaccines (Qin et al., 2016). Liu *et al.* was the first to use C3d as an adjuvant in poultry. Prior to this, research on C3d adjuvants had only been conducted on mice, cattle, and rabbits. They constructed C3d fusion proteins and conducted in vitro and in vivo experiments, discovering that C3d-P29 indeed enhances the immunogenicity of antigens, laying a foundation for the development of new vaccines (Liu and Niu, 2008). Zhao's findings also indicated that C3d, as a molecular adjuvant, could more rapidly induce an immune response, suggesting that C3d might be a novel adjuvant for coccidiosis vaccines (Zhao et al., 2017).

In addition to interleukins, tumor necrosis factors, and chemokines, other cytokines are commonly studied as molecular adjuvants (Liu et al., 2009; Li and Petrovsky, 2017). Moreover, antigen-presenting adjuvants and composite adjuvants are rapidly developing, enhancing vaccine efficacy through various mechanisms and playing indispensable roles in vaccine research.

#### Application of Antigen Targets in the Development of Various Vaccines

Live vector vaccines primarily include bacterial, viral, and protozoan types (Tang et al., 2020a). Researchers have used different vectors to express the recombinant protein AMA-1 to create live vector vaccines for immunizing chicks. Oocyst challenge experiments have demonstrated that the immune protection elicited by using *Lactococcus lactis* as vaccine vectors is more effective. Other studies found that using fowlpox virus as a vector also yielded favorable results. When the ROMs gene of *Eimeria* was introduced into the fowlpox virus and used to immunize chicks, the proliferation level of lymphocyte in the peripheral blood were significantly higher than in the control group (Yang et al., 2008). However, studies have found that adenovirus vector vaccines may be more effective than fowlpox virus vectors in preventing *Eimeria* infections, Because adenovirus vectors can infect the intestinal tissues of birds, stimulating stronger local memory immune responses (Wang and Suo, 2020).

Previous studies have demonstrated that the SO7 protein can effectively induce both humoral and cellular immunity. Building on this, Song *et al.* developed 2 DNA vaccines based on the SO7 protein. Post-immunization observations revealed that these vaccines provided notable immune protection and significantly reduced the cecal lesion scores (Song et al., 2013). It was also found that IL-2 could enhance the immune response of the SO7 protein against *E. tenella* (Song et al., 2013).

Researchers have designed a multiepitope antigen named NSLC, encoding NA4 antigen, SAG1, LDH, and other components. This antigen was then encapsulated with nanomaterials to study its immune protective effects. The findings indicated that the vaccine effectively stimulated both humoral and cellular immunity in laying hens. In addition, cross-protection against various *Eimeria* species, including *E. tenella, E. acervulina, E. necatrix*, and *E. maxima* was demonstrated (Yu et al., 2022). Proteins from the MIC family, TA4, and GM56 are also being used in DNA vaccine development. Chen and colleagues constructed DNA vaccines targeting the ROP5 and GRA15 antigens to evaluate their effectiveness against *T. gondii* (Chen et al., 2015). DNA vaccines based on ROP1 and GRA7 antigens have also shown a certain degree of efficacy in controlling toxoplasmosis (Quan et al., 2012). These protective antigens can serve as references when developing *Eimeria* vaccines, given their shared apicomplexan lineage.

In addition, chickens were immunized with a combination of *Eimeria* CD40L and IMP1 recombinant proteins. The results showed that this combination provided better immune protection compared to previous IMP1 and adjuvant combinations (Yin et al., 2015). A multivalent subunit vaccine combining TA antigen, NA4 antigen, Em8 antigen, and LDH antigen has also demonstrated significant advantages in clinical settings (Song et al., 2015a).

Antigen targets are the foundation of vaccine design, as they determine the specificity of the induced immune response. Consequently, more antigen proteins with known mechanisms of action are being used to create vaccines. For the same species, referencing known protective antigens can narrow the range of protective antigens to be screened, greatly improving vaccine development efficiency.

## Anticoccidial Drugs and Their Mechanisms of Action

### Ionophore Antibiotics

Ionophore antibiotics are commonly used to target the early developmental stages of coccidia. These drugs are known for their low toxicity, slow resistance development, broad spectrum, and high efficiency (Rutkowski and Brzezinski, 2013). Examples include salinomycin and doramectin, although some, like maduramicin ammonium, are more toxic with a narrower safety margin. These antibiotics primarily work by altering cell membrane permeability to affect normal ion transport. They interact with alkali metal ions, disrupting the cation gradient across biological membranes (Wollesen et al., 2023). This imbalance in ion equilibrium leads to a significant difference in osmotic pressure inside and outside the cell, causing the parasite cell to swell and eventually rupture (Figure 1). Although salinomycin is one of the slowest ionophore antibiotics to develop resistance, resistant strains have been reported (Flores et al., 2022). Moreover, the similar structure and mechanism of these drugs can result in cross-resistance. *Eimeria* strains resistant to monensin and diclazuril were identified using 2 forward genetic methods, providing a foundation for studying the mechanisms of drug resistance (Zhang et al., 2022, 2023b).

### Synthetic Chemical Drugs

This category includes triazines, dinitro compounds, sulfonamides, thiamine derivatives, quinolines, biguanides, pyridines, and more. Triazines, such as toltrazuril and diclazuril, mainly inhibit sporozoites and first-generation merozoites by interfering with respiratory chain enzymes, thereby affecting mitochondrial respiration and inhibiting coccidial development (Zhang et al., 2023a). Current studies have found that its metabolite Ponazuril has a stronger anticoccidial effect (Yuan et al., 2024). Common dinitro compounds like nitrotoluamide and nicarbazin target first- or second-generation merozoites. Nicarbazin, effective primarily on the fourth day postinfection, has a broad spectrum but low safety, often causing stress in chickens. Its mechanism is similar to toltrazuril. The particle size of nitrotoluamide powder affects its efficacy, with finer powders being more effective. Sulfonamides target second-generation merozoites during schizogony (Figure 1). Key drugs include sulfaquinoxaline, sulfamethazine, and sulfadimidine, with the first 2 being specialized sulfonamides for coccidiosis. Sulfaquinoxaline is often combined with amprolium or antibacterial potentiators (Campbell, 2008). The mechanism involves disrupting nucleic acid synthesis by competing with para-aminobenzoic acid for dihydrofolate synthetase, ultimately blocking nucleic acid formation (Venkatesan et al., 2023). Adding potentiators like trimethoprim can inhibit dihydrofolate reductase, significantly enhancing anticoccidial activity. Amprolium hydrochloride, a common thiamine derivative, induces thiamine deficiency in coccidia. Decoquinate, a commonly used quinoline drug, has been associated with cytochrome B mutations, which can serve as molecular markers for the rapid detection of decoquinate resistance (Hao et al., 2023). Due to the long-term irregular use of chemical anticoccidial drugs in breeding farms, coccidia has developed varying degrees of drug resistance, posing severe challenges and threats to the development of the chicken industry. This has prompted researchers to develop new drugs such as nano zinc oxide, which not only have anti coccidiosis effects but can also regulate intestinal mucosal immunity. Chlorine dioxide, in its aqueous form, shows significant resistance to *Eimeria* and is safe and nontoxic. Research has also confirmed that *Eimeria* strains exhibit resistance to halofuginone based on the resistance mechanism observed in *Plasmodium*. Additionally, it has been found that halofuginone resistance is closely related to A1852G and A1854G mutations in the ETH2_1020900 gene. This finding provides a method and foundation for studying the resistance mechanism (Sun et al., 2023a, b).

### Traditional Chinese Medicine

With increasing emphasis on Traditional Chinese Medicine (**TCM**), TCM formulations have emerged in treating many diseases, especially those developing resistance to chemical drugs. The same principles apply to animal husbandry. The previous study has summarized various herbs that can stimulate both cellular and humoral immune responses, as well as herbs with significant anti-coccidial effects. These herbs not only enhance the immunity of the chicks but also reduce lesion scores and oocyst shedding (Muthamilselvan et al., 2016; Rizwan et al., 2022). Other formulations like *Magnolia officinalis* compound, tea saponin, and paper mulberry leaf powder have also shown some preventive effects against chicken coccidiosis.

## Sporogony Stage-Based Control Agents and Their Mechanisms of Action

### Biological Research on Sporogony Regulation

Recent studies have identified the APETALA2 (AP2) family as potential key biological regulators (Cai et al., 2023). Initially discovered in plants, where they play crucial roles in development, these proteins were later identified in Apicomplexan parasites and named Apicomplexan AP2 (**ApiAP2**) proteins (Jeninga et al., 2019). AP2 proteins are essential for gene expression in Apicomplexan parasites. The AP2XII-2 gene has been found to play a crucial role in the several development stages of *Plasmodium* and *T. gondii* (Srivastava et al., 2023; Fan et al., 2023; Painter et al., 2011). There are 53 proteins containing AP2 domains in the *Eimeria* genome have been identified (Chen et al., 2023). The authors further studied the expression patterns of AP2 transcription factors, analyzing expression profiles at different developmental stages. They constructed overexpression and knockout models for the sporozoite stage-specific ApiAP2 gene ETH2_0411800 and analyzed its effect on *Eimeria* development. However, their findings suggest that this gene may not play a significant role in the development of *Eimeria*. Another ApiAP2 gene, ETH2_0940300, is theoretically critical for cell division and the regulation of parasite development, but the establishment of a knockout strain for this gene has not yet been successful, preventing experimental confirmation of its function (Chen et al., 2023). In another study, ApiAP2 gene named ENH_00027130 were cloned from the cDNA of *E. necatrix* second-generation merozoites, and subsequently expressed by *E. coli*. The authors analyzed the subcellular localization of the EnApiAP2 protein both *in vivo* and *in vitro* using Western blot and indirect immunofluorescence assays. They found that the EnApiAP2 protein localized in the parasite's nucleus, suggesting it might be a nuclear transcription factor. The study also indicated that EnApiAP2 might regulate asexual reproduction rather than sexual reproduction, providing a foundation for further exploration of the role of EnApiAP2 in the *Eimeria* life cycle (Cai et al., 2023).

### Disinfectants for Coccidia Oocysts

Infectious oocysts are the primary source of transmission for coccidia. Disinfectants can inhibit or kill these oocysts, but mild formulations are often ineffective, while highly potent disinfectants can be toxic to animals and farm workers. Thus, finding efficient, low-toxicity disinfectants is crucial. In 1997, Williams proposed that ammonia had a better effect on killing coccidia (Williams, 1997). Subsequently, some researchers tested the inhibitory and lethal effects of 17 commonly used disinfectants on *Eimeria* oocysts. They found that 8% ammonia had the best effect, followed by NaOH, peracetic acid, phenols, formaldehyde, and toltrazuril, which partially inhibited oocyst sporulation. They emphasized that the concentration and exposure time of ammonia must be strictly controlled to ensure thorough eradication of oocysts. In China, it has confirmed that compound para-chloro-meta-cresol is more effective than phenol-based disinfectants and could replace ammonia as a new standard disinfectant (Figure 1). Ammonia, while effective, strongly irritates the respiratory system, skin, and eyes, and inadequate ventilation after disinfection could cause respiratory or skin diseases in poultry farms. Through a comparison of 10 chemical disinfectants, the results showed that acetic acid, benzene, and xylene have significant inhibitory effects on coccidia oocysts (You, 2014). In 2017, a study discovered that 10% formalin and 70% ethanol exhibited a superior inhibitory effect on coccidian oocysts (Gadelhaq et al., 2018). Another study found that a 5% potassium hydroxide disinfectant provided better disinfection results compared to allicin and alcohol allicin (Abd-ELrahman et al., 2022). In addition, ozone, a disinfectant used to treat waste water, also plays a significant role in preventing the sporulation of coccidia oocysts (Baumann et al., 2024).

### Mechanisms of Action of Coccidia Oocyst Disinfectants

Coccidial infection occurs when poultry ingest sporulated oocysts, which are then infectious. Inhibiting the sporulation process or killing already sporulated oocysts can significantly reduce coccidial infection. The oocyst wall of *Eimeria* oocysts is generally composed of 2 or more layers. The outer layer is composed of chitin like substances and quinine-tanned proteins, while the inner layer is composed of approximately 30% lipids and 70% proteins (Abd-ELrahman et al., 2022). The oocyst wall is highly electron-dense, providing strong resistance to external damage and making water-soluble substances difficult to penetrate. However, it allows gas exchange and the passage of lipid-soluble substances and small molecules. The oocyst wall is also sensitive to high heat; boiling water and steam can easily kill it. Therefore, lipid-soluble or small-molecule disinfectants are more effective against coccidia oocysts, and heated disinfectants may enhance this effect. There are few acid or alkaline disinfectant products specifically designed to kill oocysts. Effective alkaline disinfectants are highly corrosive and can cause harm if used improperly. Weak acid or alkaline disinfectants commonly used in farms ionize easily and cannot penetrate the oocyst wall effectively, resulting in weak disinfectant action. Chlorine dioxide, a high-efficiency disinfectant for livestock and poultry, has strong adsorption and penetration capabilities against microbial cell walls. It effectively oxidizes sulfhydryl-containing enzymes within cells and can rapidly inhibit protein synthesis to destroy microorganisms. In addition, the nitric oxide donor S-nitroso-glutathione was found to inhibit coccidia oocysts, but its effectiveness was limited to the early stage of sporulation (Li et al., 2008, 2010).

### Natural Products against Sporulation of Coccidia Oocyst

Essential oils extracted from leaves, stems, fruits, and roots through pressing, extraction, or distillation have notable antibacterial activity and serve as potential green alternatives for coccidiosis prevention (Muthamilselvan et al., 2016). For example, essential oils from Artemisia, tea tree, and thyme have oocyst-killing activity, but the effect of essential oil combination is different (del-Cacho et al., 2010; Fatemi et al., 2015; Isakakroudi et al., 2018). Pine bark extract and garlic essential oils can reduce the sporulation of the *Eimeria* oocysts, lower intestinal lesion scores, reduce the spread of chicken coccidiosis, and improve broiler performance (Molan et al., 2009; Wunderlich et al., 2014). Artemisia absinthium has been verified strong anti-coccidial effects using systematic review and meta-analysis, especially in inhibiting the sporulation of coccidian cysts (Hezil et al., 2024). The active ingredients in panduratin oil and basil oil could kill oocysts, reducing the prevalence of coccidiosis and aiding in its prevention (Jitviriyanon et al., 2016). A series of methods were used to evaluate the opuntia ficus-indica Flower Extracts on *Eimeria* oocysts and found that extracts can effectively inhibit sporulation and disrupt the morphology of *Eimeria* oocysts (Amrane-Abider et al., 2023). In addition, alcoholic vitis vinifera leaf extracts, allicin and alcoholic garlic extract also have a significant effect on disrupting the morphology of *Eimeria* oocysts (Abd-ELrahman et al., 2022; Murshed et al., 2023). Due to their lipid solubility and low molecular weight, essential oils quickly diffuse into the parasite through host cell membranes, disrupting the parasite's cell membrane structure, causing leakage of cell contents, and leading to death.

## Perspective

Coccidiosis poses a significant threat to modern poultry farms due to its widespread nature and ability to infect chickens regardless of season, age, or breed. The disease primarily spreads through sporulated oocysts present in the environment. Using disinfectants during the vacant periods of the chicken rearing cycle to eliminate oocysts is a strategic approach to prevent the spread of coccidiosis. However, conventional disinfectants are generally ineffective against coccidia oocysts. While oocysts are particularly sensitive to ammonia, its use presents safety risks for animals, facilities, and personnel. The misuse of antibiotics has led to drug resistance and residue issues, resulting in the banning of many anticoccidial drugs. Moreover, feeding chickens anticoccidial drugs to reduce the incidence of coccidiosis has its limitations.

Once ingested, sporulated oocysts initially undergo partial digestion in the chicken's gizzard, then fully release and proliferate extensively in the intestines. The subsequent oocysts excreted in feces are not immediately infectious. If the development of these oocysts could be inhibited during the period from excretion to sporulation, preventing them from successfully sporulating, coccidiosis would not spread. However, current methods of environmental disinfection, administering anticoccidial drugs, and vaccine immunization all have certain variabilities. Therefore, there is an urgent need for new, effective, and safe anticoccidial methods.

Advances in bioinformatics and its application in parasitology, along with the establishment of CRISPR/Cas9 gene editing technology in *Eimeria*, offer promising avenues for deeply analyzing the developmental regulation mechanisms of *Eimeria* sporogony (Hu et al., 2020; Tang et al., 2020b; Chen et al., 2021). Identifying key regulatory genes and developing vaccines based on sporogony-deficient strains are crucial directions for future coccidiosis vaccine development (Reid et al., 2014). Additionally, understanding how *Eimeria* responds to external environmental stimuli to initiate sporogony can facilitate the development of highly effective, *Eimeria*-specific sporogony inhibitors. This knowledge can lead to novel products that significantly improve the control of coccidiosis.

## DISCLOSURES

The authors declare that the research was conducted in the absence of any commercial or financial relationships that could be construed as a potential conflict of interest.
